# Heart Failure Associated With Ventricular Septal Defect, Mitral Valve Prolapse, Non-stenotic Bicuspid Aortic Valve, and Patent Foramen Ovale

**DOI:** 10.7759/cureus.22020

**Published:** 2022-02-08

**Authors:** Antoine El Khoury, Elham Lagha, Zela Maria Butchakdjian, Mary-Joe Touma, Chady Kharrat, Assaad Maalouf

**Affiliations:** 1 Cardiology, University of Balamand, Beirut, LBN; 2 Internal Medicine, Lebanese University, Beirut, LBN; 3 Internal Medicine, University of Balamand, Beirut, LBN; 4 Cardiology, Saint George Hospital University Medical Center, Beirut, LBN

**Keywords:** global longitudinal strain, mitral valve prolapse, bicuspid aortic valve disease, patent foramen oval, ventricular septal defect (vsd), heart failure with reduced ejection fraction

## Abstract

Ventricular septal defect (VSD) and bicuspid aortic valve (BAV) are the two most common congenital heart defects. BAV may occur sporadically or in association with other cardiac malformations. VSDs have decreased incidence in the adult population due to spontaneous closure. Mitral valve prolapse (MVP) and patent foramen ovale (PFO) can be associated with these conditions but the simultaneous association of these defects has never been reported in the literature. We report the case of a 35-year-old male patient with persistent VSD, BAV, and PFO associated with new-onset heart failure and MVP. We aim to study the association between the coexistence of structural heart malformations and the increased risk of heart failure.

## Introduction

Bicuspid aortic valves (BAV) have an incidence of 1%-2% in the general population [1]. Ventricular septal defects (VSD) can occur as an isolated finding or in association with other cardiac anomalies: atrial septal defects, pulmonic stenosis, and patent ductus arteriosus [2]. BAVs present as the most common congenital heart anomaly in adults, with VSD being the second most common [3]. The co-occurrence of BAV and VSD has been previously reported [4]. Perturbations in the endocardial cushion remodeling process have been associated with the development of both BAV and VSD [4]. Owing to their shape, BAVs are generally more prone to increased hemodynamic stress thus predisposing the patient to heart failure which requires regular monitoring [5]. Missed diagnosis of patients with BAVs usually occurs as a consequence of failure to present earlier in life because it remains asymptomatic until late in the disease course. Patients usually present between ages 20 to 40 for evaluation of a diastolic heart murmur [5]. VSDs can close spontaneously, but persisting ones, if uncorrected, can lead to hemodynamic instability due to the shunting of the blood from left to right ventricle (RV) [6]. MVP which can be familial or sporadic is caused by the displacement of one of the leaflets into the left atrium. It is diagnosed by echocardiography and can be a cause of mitral regurgitation [7]. Patent foramen ovale (PFO) is a remnant of the fetal circulation and is a congenital opening in the intra-atrial septum. It can lead to right-to-left shunting and cryptogenic strokes [8].

## Case presentation

**Figure 1:**
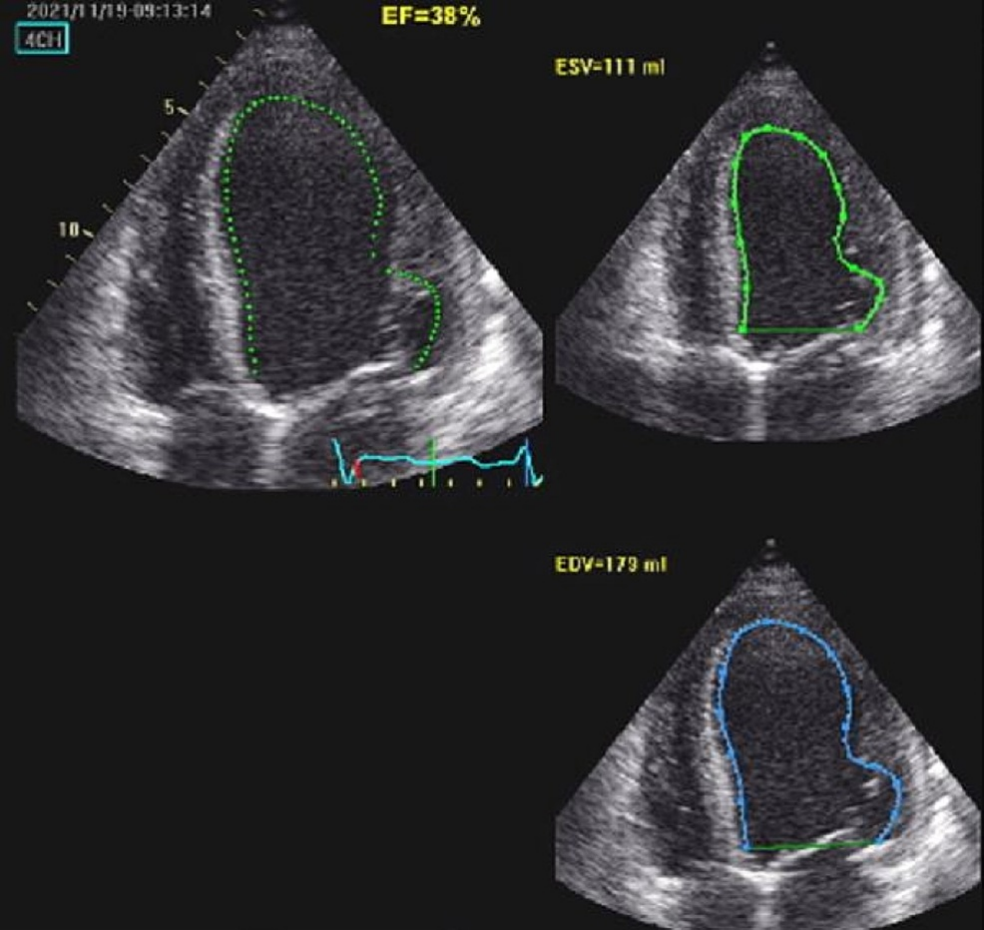
Four-chambers view with auto-ejection fraction (EF) function measuring EF of 38%.

**Figure 2:**
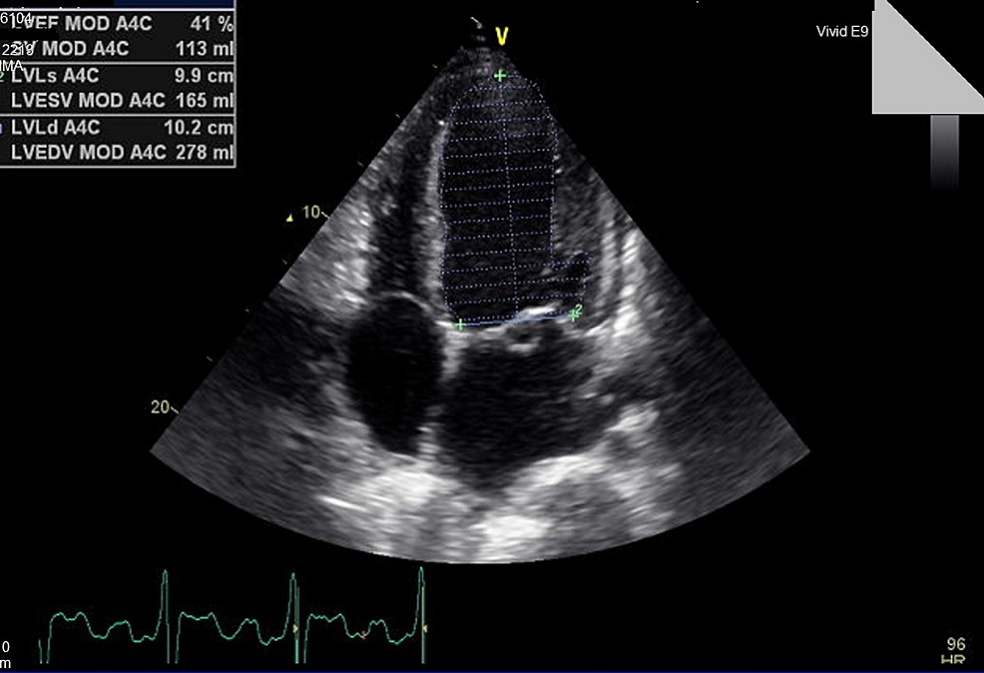
Biplane methods estimating the cardiac ejection fraction with a value of 41%.

**Figure 3:**
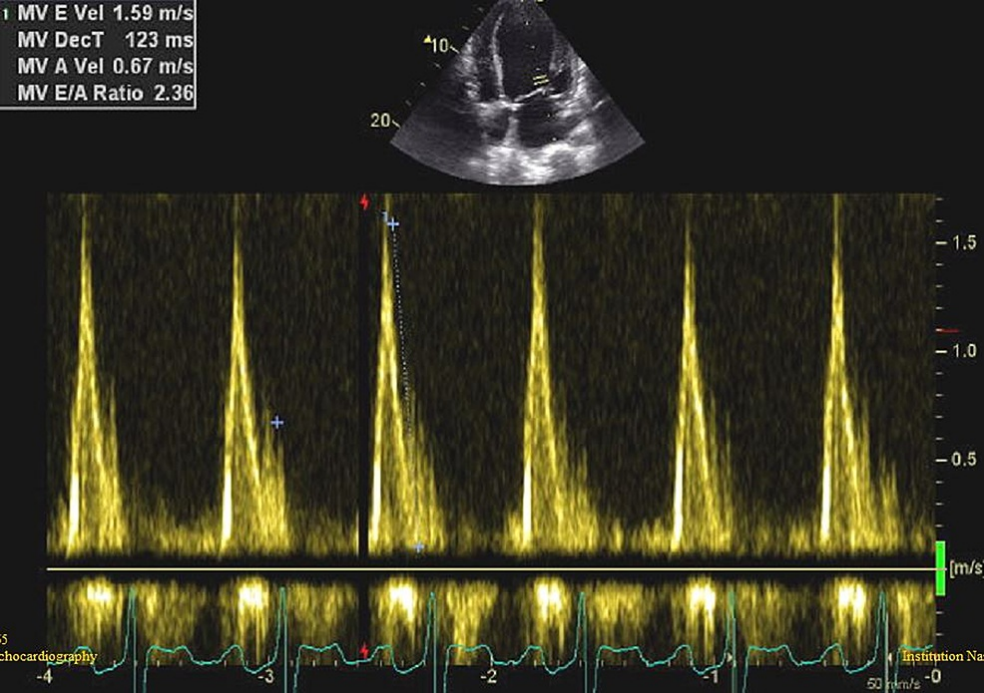
Pulse wave Doppler image showing signs of restrictive pattern with high E waves velocities.

A 35-year-old male with a history of BAV and a perimembranous VSD without hemodynamic consequences presented for follow-up echocardiography. The family history is negative for similar structural heart diseases but is positive for ischemic heart diseases in both parents. The last transesophageal echocardiography performed two years prior to presentation showed normal LV systolic and diastolic function and wall motion, a BAV with mild aortic regurgitation but no stenosis. A small perimembranous VSD of 0.7 cm was noted with a minimal shunt. The RV was normal with normal pulmonary artery systolic pressure. A foramen ovale was noted but the agitated saline test showed very minimal passage of bubbles to the left-sided cavities. There was no visible thrombus and the rest of the valves were normal. The patient was stable since the examination was performed but lost to follow up. Two years later, the patient started progressively complaining of new symptoms including dyspnea that started on exertion and progressed to less than ordinary physical activity and was partially relieved by rest condition attributable to New York Heart Association (NYHA) class three. There was no jugular venous distention but the presence of bilateral basal lung crackles and mild pitting edema of the lower limbs were seen on physical exam. The onset of the edema was associated with the appearance of symptoms. The new transthoracic echocardiography showed evidence of new-onset moderately dilated left ventricle (LV) with moderate systolic dysfunction (ejection fraction (EF) = 40%) (Figures 1-2) (Videos 1-3). There was global hypokinesia and severe diastolic dysfunction (grade IV) and a restrictive pattern as converted by the pulse wave Doppler studies (Figure 3). A proBNP was done and was elevated with a level of 2240. 

**Video 1:** Parasternal long axis view of the heart showing new-onset reduced systolic function.

**Video 2:** Parasternal short axis view of the heart showing new-onset reduced systolic function.

**Video 3:** Apical four chambers view of the heart showing new-onset reduced systolic function and prolapse of the A2 leaflet of the mitral valve.

**Figure 4:**
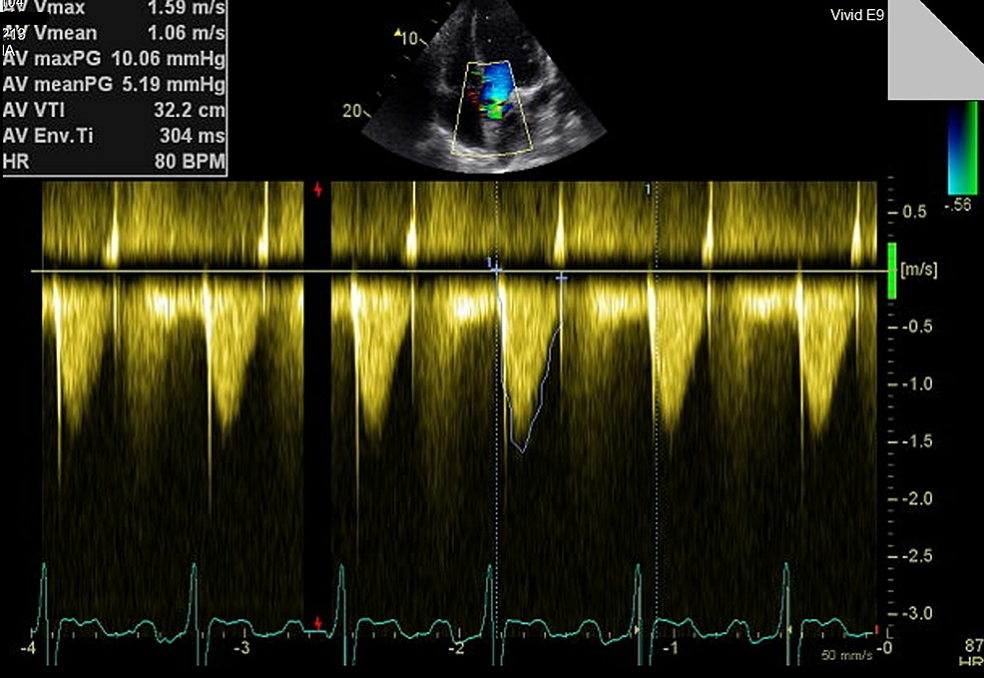
Continuous wave Doppler across the aortic valve showing normal velocity and normal mean gradient.

**Figure 5:**
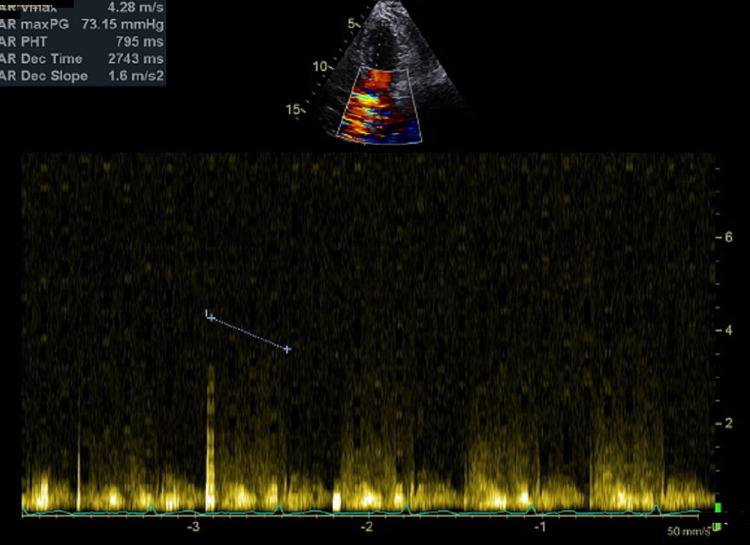
Continuous wave Doppler across the aortic valve measuring pressure half time showing mild aortic regurgitation.

**Figure 6:**
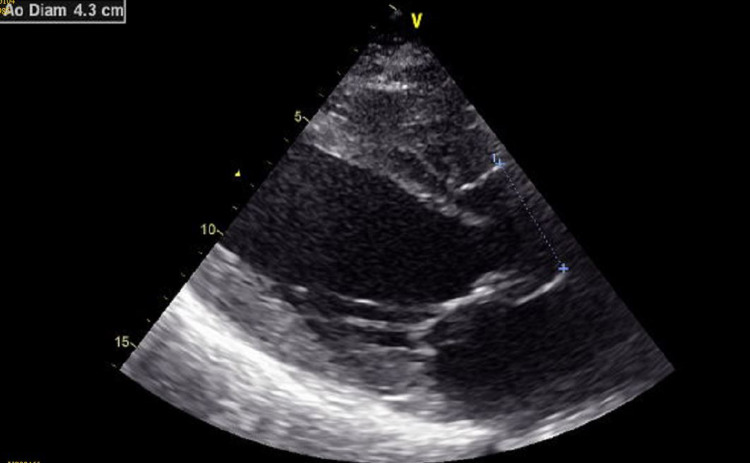
Parasternal long axis view showing dilatation of the ascending aorta.

**Figure 7:**
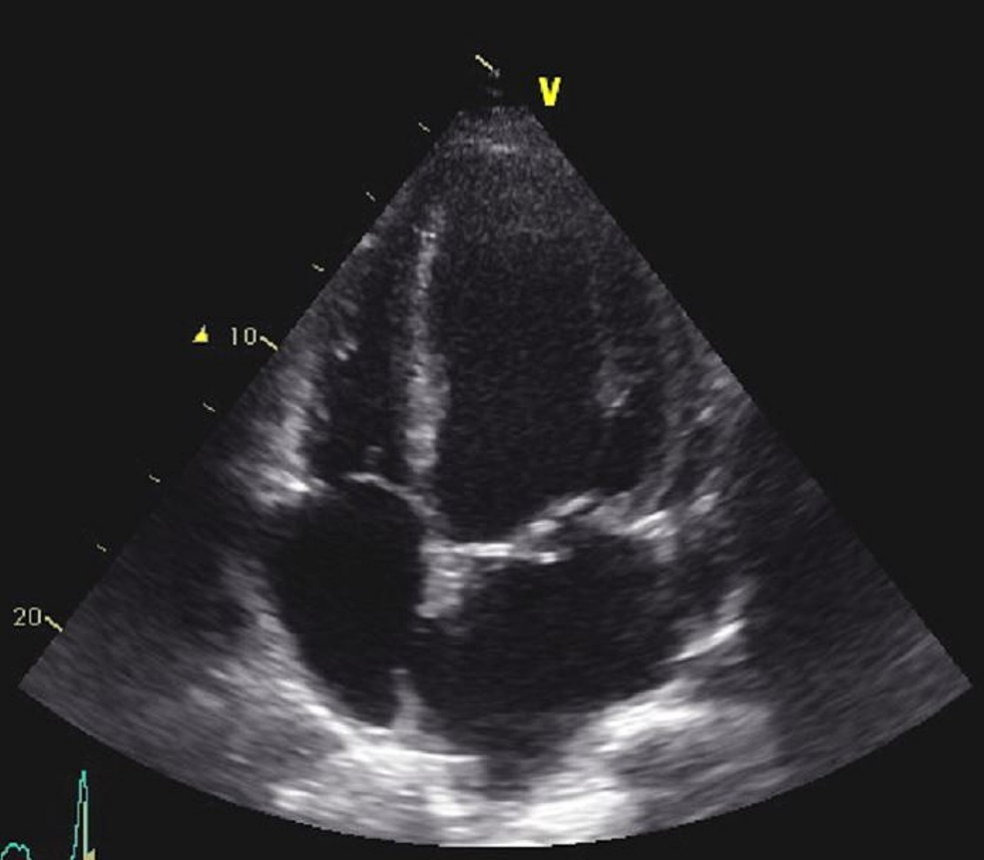
Apical four-chamber view of the heart showing mitral valve prolapse.

**Figure 8:**
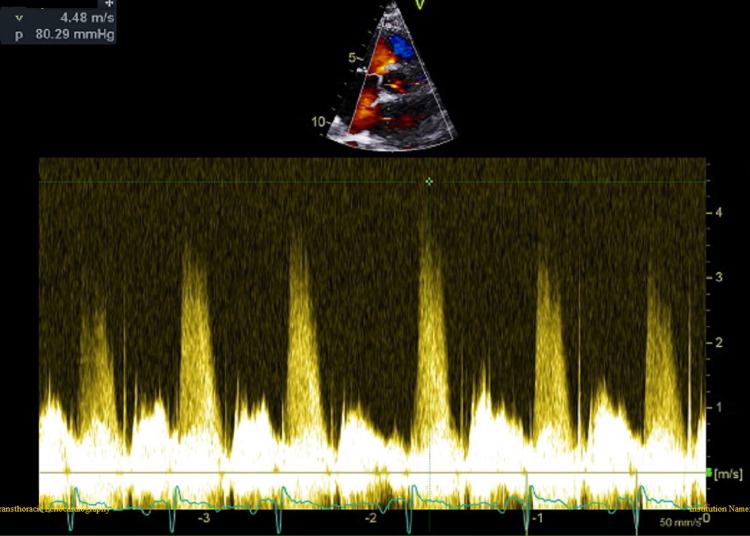
Continuous wave Doppler aligned with ventricular septal defect showed an LV-RV gradient of 80 mmHg. LV: left ventricle; RV: right ventricle.

The aortic valve was bicuspid as confirmed by the previous examination but there was no progression of the regurgitant jet, only mild regurgitation with a pressure half time of 795 milliseconds and there was no valvular stenosis with a Vmax of 1.59 m/s and a normal mean gradient (Figures 4-5) (Video 4). There was aneurysmal dilation of the ascending aorta at the level of the sinus of Valsalva (43 mm), as can be seen by Figure 6, and evidence of new-onset mitral valve prolapse of the A2 leaflet with mild mitral regurgitation and no stenosis (Figure 7) (Video 3). The VSD was stable in size of 7 mm with mild left-to-right shunting (Video 5). The continuous wave Doppler aligned with the maximum jet of the VSD and showed left to RV gradient of 80 mmHg (Figure 8). The patient had no left ventricular outflow tract obstruction, the RV systolic pressure was calculated using the equation right ventricular systolic pressure (RVSP) = systolic blood pressure -4V^2. The patient systolic blood pressure was 110 mmHg, this will give an RVSP of 110 minus 80 which is equal to 30 mmHg. 

**Video 4:** Parasternal short axis view of the heart showing bicuspid aortic valve.

**Video 5:** Apical three-chambers view of the heart showing evidence of ventricular septal defect with left to right shunting.

**Figure 9:**
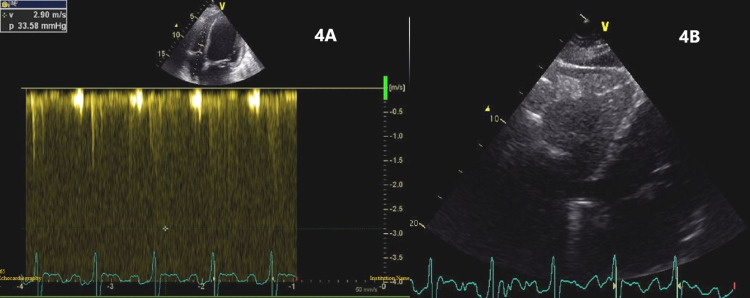
Continuous wave Doppler across the tricuspid valve showing mildly elevated right ventricular pressure (A) and the inferior vena cava was not dilated (B).

**Figure 10:**
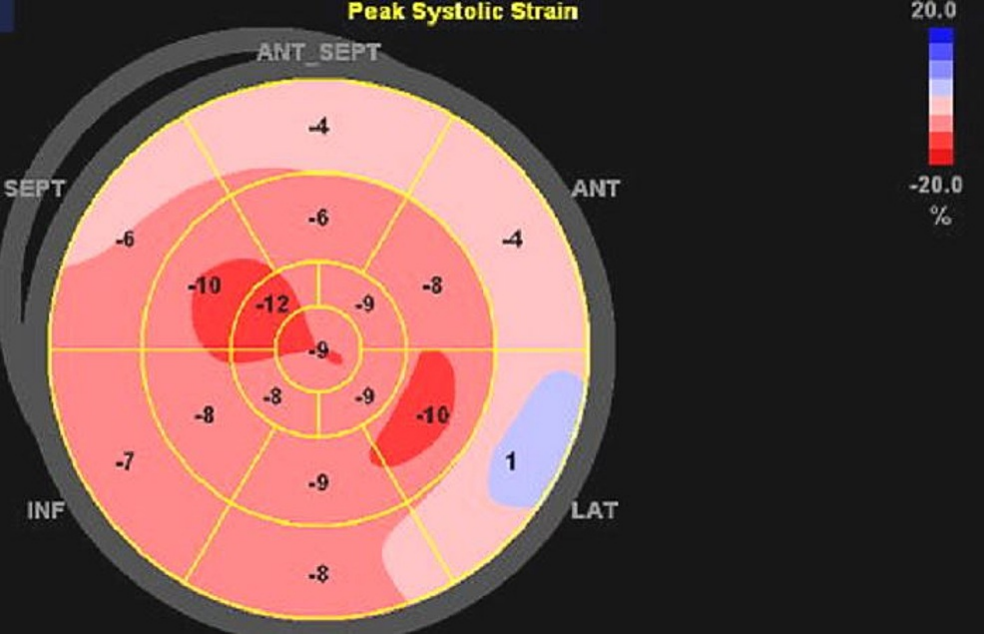
Global longitudinal strain of the heart showing globally depressed function with an average of -7.2.

The RV systolic pressure was only mildly elevated without significant RV dilatation and the inferior vena cava was non-dilated with normal respiratory variations (Figure 9). The global longitudinal strain was depressed in all heart segments with an average value of -7.2 and the most affected segment being the lateral wall with a value of +1 (Figure 10). The patient had the association of BAV, VSD, mitral valve prolapse of the A2 leaflet, and PFO. 

The patient was started on betablockers with bisoprolol 5 mg once daily and sacubitril/valsartan combination to treat his heart failure with EF<40% and was scheduled to perform a cardiac MRI to assess for underlying cardiomyopathy but the patient declined did not follow up. Closure of the VSD was not recommended for the moment since the LV-RV gradient is not very significant and the RV is not dilated with borderline systolic pulmonary artery pressure. The aortic valve was scheduled for observation to follow up on the regurgitation and the ascending aorta diameter. 

## Discussion

Congenital and structural heart defects are not uncommon in the general population and affect 1% of the total births per year [9]. Different associations between structural heart defects have been previously reported, however, the co-occurrence of non-stenotic BAV, together with VSD, MVP, and PFO with new-onset heart failure seen in our patient appears to be the first of its kind.

A study evaluating the genetic inheritance of structural heart defects on a population of mice revealed that genes on chromosome X code for the filamin proteins which are responsible for cardiac and vascular development [7]. Mutations in this protein resulted in a combination of structural heart defects in mice [7]. Associations between atrial septal defects, VSDs, aortic arch defects, abnormal vascular permeability, and MVP were noted in animals with mutated gene coding for the filamin protein. A similar mutation could lead to the association of defects in humans [7]. 

BAV is the most common adult congenital heart defect. It is often associated with both dilation and aneurysm of the ascending aorta [3]. BAVs are associated with dilation of the ascending aorta in 50% of cases [10], furthermore, they are usually correlated with significant aortic stenosis and aortic dilation [10]. Membranous VSD, which was found in our patient, is the most common of the several types of VSD [11].

The echocardiography performed on our patient one year prior to presentation showed the presence of these structural defects without the presence of heart failure. The patient was advised for repair of the VSD, however, he was lost to follow-up. It is important to stress the frequent echocardiography follow-up from a young age in order to prevent irreversible detrimental effects [12]. 

Our patient presented two years later with new-onset debilitating heart failure symptoms. The rapid onset of heart failure, which was associated with impaired global longitudinal strain on echocardiography, made us think of an associated cardiomyopathy such as sarcoidosis or amyloidosis. On echocardiography, there was evidence of left-to-right shunting, however, it was not associated with increased right-sided pressures. In order to rule out ischemic causes of his new-onset heart failure due to a family history of premature coronary artery disease, coronary angiography was performed and showed no evidence of ischemic heart disease.

Following the abrupt deterioration of the patient’s condition, we postulate that all four mentioned structural adult heart defects work synergistically to hasten the onset of severe heart failure. Furthermore, we also hypothesize that the co-occurrence of these etiologies has unveiled and accelerated the onset of the underlying masked cardiomyopathy that was not evident on the old echocardiography.

Heart failure (as confirmed by auto-EF, biplane method, and global longitudinal strain) in our patient could be related to a different underlying condition, not necessarily from his structural heart defects. This is due to the fact that the right-sided pressures are not very elevated, meaning that the hemodynamic consequences of the VSD and the left-to-right shunt are not causing significant RV dilatation or strain. In addition, BAV has a relatively normal function in our patient without significant stenosis and regurgitation making it a less likely trigger to the new-onset heart failure. Accordingly, the patient was advised to do a cardiac MRI in order to look for an alternative etiology for his heart failure. 

## Conclusions

The association of structural heart defects has been described in the literature and most of these defects are diagnosed incidentally during adulthood. However, the occurrence of four structural defects in the same patient (membranous VSD, BAV, MVP, and PFO) is not described in the literature. Heart failure is associated with congenital heart defects and the combination of these defects could worsen or accelerate the onset of heart failure symptoms at a young age. In case of association of defects, and in case of hemodynamic significance, it might be better to treat the defects instead of just observing in order to prevent complications including heart failure.
